# Protective Effects of Probiotic Consumption in Cardiovascular Disease in Systemic Lupus Erythematosus

**DOI:** 10.3390/nu11112676

**Published:** 2019-11-05

**Authors:** Néstor de la Visitación, Iñaki Robles-Vera, Marta Toral, Juan Duarte

**Affiliations:** 1 Department of Pharmacology, School of Pharmacy, Universidad de Granada, 18071 Granada, Spain; nestorvp@correo.ugr.es (N.d.l.V.); roblesverai@ugr.es (I.R.-V.); jmduarte@ugr.es (J.D.); 2 Gene Regulation in Cardiovascular Remodeling and Inflammation Group, Centro Nacional de Investigaciones Cardiovasculares Carlos III (CNIC), 28029 Madrid, Spain; 3 Ciber Enfermedades Cardiovasculares (CiberCV), 28029 Madrid, Spain; 4 Instituto de Investigación Biosanitaria de Granada (Ibs.GRANADA), 18012 Granada, Spain

**Keywords:** immune response, gut microbiota, hypertension, endothelial dysfunction, nephritis

## Abstract

The prevalence of renal and cardiovascular disease (CVD) in patients with systemic lupus erythematosus (SLE) is higher than in general populations. Recently, a causal role of gut microbiota on the development of immune responses in SLE has been described. Probiotic consumption changes the composition of gut microbiota, preventing SLE progression. The aim of this review is to explore the role of the gut microbiota in the development of renal and cardiovascular disease in SLE and how probiotics could be a therapeutic option. Despite strong evidence on the beneficial effects of probiotics in the development of autoimmunity and nephritis in SLE, only a few studies described the protective effects of *Lactobacillus* in important risk factors for CVD, such as endothelial dysfunction and hypertension in mice. The preventive effects of probiotics in renal and CVD in humans have not been established yet.

## 1. Introduction

Systemic lupus erythematosus (SLE) is a chronic autoimmune disease characterized by abnormally functioning B lymphocytes [1], which promote an exacerbated production of autoantibodies that trigger the formation and deposition of immune complexes that damage many organs and tissues [2]. While the causes are not known, it is widely considered that SLE is the consequence of the effects of environmental factors in genetically predisposed individuals, leading to the disruption of self-tolerance and to the activation/increase in innate immune cells and autoreactive lymphocytes [3].

**Figure 1:**
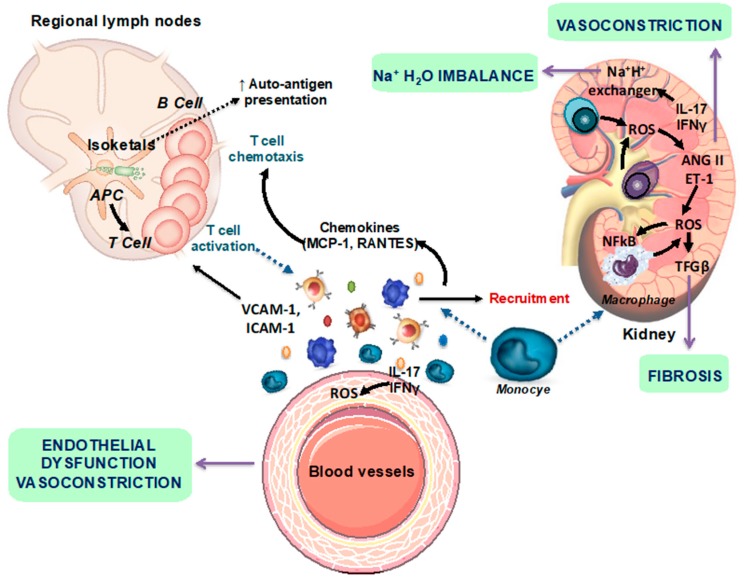
Scheme representing the autoimmune mechanisms involved in the development of renal and vascular changes. AngII, angiotensin II; ET-1, endothelin-1; IFN-γ, interferon-γ; IL-17a, interleukin 17a; NF-kB, nuclear factor-kB; ROS, reactive oxygen species; Treg, T regulatory (modified from [10]).

SLE is associated with renal and cardiovascular disease (CVD) [4]. In particular, hypertension is thought to be the greatest risk factor for cardiac events in lupus populations [5]. In fact, numbers ranging from 33% to 74% of SLE patients have been described to present hypertension [6]. Nevertheless, there has been little exploration of the pathophysiological mechanisms that promote SLE hypertension [7]. Several studies using female NZB/WF1 mice, a spontaneous model of SLE that mimics human disease and develops hypertension, have demonstrated that multiple factors contribute to the pathogenesis of hypertension, including inflammatory cytokines, tumor necrosis factor (TNF)-α, and oxidative stress. These mediators, which contribute to local inflammation and the subsequent renal and vascular dysfunction [8,9,10], are likely downstream of the initial immune system dysregulation [11]. Hypertension is associated with the infiltration of immune cells into the adventitia and periadventitial fat, as well as the activation of T cells that release proinflammatory cytokines such as interleukin (IL)-17a, interferon (IFN)-γ, and TNF-α [12,13]. However, T, but not B, cells play a key role in the modulation of blood pressure in response to angiotensin II-mediated hypertension [12]. Hypertension in female NZB/WF1 mice is associated with low plasma renin and is not sensitive to salt. In this mice model of SLE, treatment with anti-CD20 antibody, which lowered the percentage of B cells in the spleen and the quantity of anti-double-stranded (anti-dsDNA) antibodies in plasma, prevented the development of hypertension [14]. This study highlights the importance of B cells in the progression of SLE hypertension. However, the exact role of hyperactive T and B lymphocytes, both central to the development of autoimmune disorders, in the pathogenesis of hypertension remains unclear. Several studies suggest that immune cells, oxidative stress and inflammation are linked in a self-perpetuating cycle, which significantly contributes to the renal damage and vascular disease associated with hypertension in SLE [8]. T cell activation occurs in secondary lymphoid organs and locally within target organs, specifically the kidney and blood vessels. Neoantigen presentation is enhanced by oxidative stress in the antigen-presenting cells through isoketal production that enhance inflammatory responses. The effector mechanisms evoked by cytokines include fibrosis, vasoconstriction and Na^+^/H^+^ imbalance (Figure 1).

However, whereas traditional risk factors associated with atherosclerosis, including hypertension, are present in lupus patients, they do not fully explain the high rate of ischemic events so far reported [15]. This implies that other factors inherent to disease itself contribute to the cardiovascular burden seen in these patients, such as, clinical, genetic, and immunological contributors. Disease duration, chronic organ damage and disease activity are important clinical factors for CVD development in the setting of SLE. Genetic data on lupus-related atherosclerosis are rather scarce. The presence of the rs10181656(G) signal transducer and activator of transcription factor 4 (STAT-4) allele or the prevalence of the minor A allele and the AA genotype of the rs12583006 B-cell activating factor (BAFF) variant conferred increased susceptibility for arterial events and ischemic cerebrovascular disease in the context of SLE. The imbalance between endothelial damage, as a result of several insults (deposition of oxLDL, autoantibodies, type I interferons, neutrophil extracellular traps), and atheroprotective mechanisms, seems to be a central event for immunological contributors to atherosclerosis in the setting of lupus [16].

Nowadays, the treatment and management of SLE is primarily based on non-steroidal anti-inflammatory drugs, glucocorticoids, hydroxychloroquine, and immunosuppressive agents [17]. Progress in the treatment of SLE has resulted in a significant improvement in prognosis. Nonetheless, SLE management is challenging because of the adverse effects of conventional therapies and the occurrence of refractory disease. In fact, corticosteroids and azathioprine therapies have been associated with an increased rate of CVD in lupus patients. Therefore, there is an imperative need for new treatment strategies that would allow us to treat renal and cardiovascular disorders in lupus patients without compromising their health state.

In this way, different studies have demonstrated that gut microbiome dysbiosis has been associated with autoimmune diseases such as type 1 diabetes, inflammatory bowel disease, rheumatoid arthritis, and multiple sclerosis. However, little is known on the role of gut microbiota in SLE in both animal models and humans [18,19,20]. Thus, the inclusion of probiotic supplementation in cardiovascular risk management should be considered. In fact, supplementation for a long period with multispecies probiotic mixtures exerts a favorable and dose-dependent effect on liver function and lipid profile in the rat model and may also have a favorable influence on cardiovascular impairments [21].

Furthermore, manipulation of the gut microbiota may lead to the development of novel therapies in SLE patients.

Specifically, we review the changes in the microbiota associated with SLE and discuss current knowledge on the impact of probiotics with immune-modulatory properties in the development of renal and cardiovascular disease on animal models and on human lupus patients as novel therapies.

## 2. Gut Microbiota and SLE

The mammalian microbiome consists of a unique set of microorganisms (i.e., bacteria, archaea, fungi, and viruses) associated with various niches in and on the body. The gut microbiota is dominated chiefly by *Firmicutes* and *Bacteroidetes*, and, to a lesser extent, by *Actinobacteria*, *Proteobacteria*, *Synergistetes*, *Verrucomicrobia* and *Fusobacteria.* However, gut microbiota constantly adapts to lifestyle modifications, such as diet, hormones and exercise [22]. In recent years, several parameters of health and disease have been found linked to shifts in the human gut microbiome.

### 2.1. Dysbiosis in SLE Patients

Human healthy gut microbiota is difficult to identify taking into account its interindividual variability and plasticity. However, ecological parameters of microbial stability, such as richness and diversity, are often used as indicators of gut health, since they are inversely associated with chronic diseases [23]. Lupus has been associated with several changes in gut microbiota (Table 1), which could be correlated with the manifestations of the pathology. However, those alterations are not fully understood yet, because there are variances among humans from different countries [24]. Contradictory results linking SLE to shifts in ecological parameters, such as richness and diversity, have been described. In fact, He et al. [24] showed significantly lower alpha diversity (Phylogenetic Diversity (PD) whole tree and observed species) in SLE patients, without differences in Shannon or Simpson. According to this, Li et al. [25] described a reduction in diversity metrics as Chao and observed species in SLE patients. In contrast, Hevia et al. [18] did not find significant differences in any alpha diversity measures (Chao, PD whole tree, observed species, Shannon, and Simpson indexes). Maybe these discrepancies could be explained by the influence of sex, age, progression of the disease and genetics background.

When the microbiota has been more deeply analyzed, the authors have found certain differences that could explain its role in SLE. At the phylum level, there seems to be a clear increase in *Bacteroidetes, Actinobacteria* and *Proteobacteria* and shrinking of *Firmicutes* in SLE patients, with these changes stable being between patients of different countries [24]. Recently, the reduction in *Tenericutes* and a rise in *Fusobacteria* in SLE has been described [25]. Overall, these changes are characterized by an intestinal dysbiosis associated with an alteration in the *Firmicutes/Bacteroidetes* (F/B) ratio (increase in *Bacteroidetes* and reduction in *Firmicutes*), although some authors have not found significant differences in this parameter between SLE and healthy patients [26]. At the family level, SLE patients from different studies presented varied results. He et al. [24] has described how SLE patients from Spain showed a depletion of *Lachnospiraceae* and *Ruminococcaceae* and an enrichment of *Bacteroidaceae* and *Prevotellaceae* but only *Prevotellaceae* showed significant increases in SLE patients from China [24]. Furthermore, *Rikenellaceae*, *Streptococcaceae*, *Lactobacillaceae* and *Megasphaera* might increase in SLE. These changes at the family and phylum levels are joined to changes at the genera level. At the genera level, we can highlight alterations in *Pseudobutyvibrio*, *Dialister*, *Lactobacillus*, *Bifidobacterium*, *Mollicutes*, *RF39*, *Faecalobacterium*, *Cryptophyta* and *Roseburia*, which are depleted in SLE patients. On the other hand, the genera *Rhodococcus*, *Eggerthella*, *Klebsiella*, *Prevotella*, *Eubacterium*, *Flavonifractor* and *Blautia* are enriched in this pathology [24,25,26]. The analysis of intestinal microbiota from patients with the pathology in the remissive stage found an increase in the *Bifidobacterium* genus [25]. At the species level, certain species were elevated in the gut microbiota of SLE [25], specifically, *Streptococcus anginosus*, *Lactobacillus mucosae*, and *Veinella dispar*. These changes appeared accompanied by a reduction in *Ruminococcus gnavus and Bacteroides uniformis* [27].

It might be an important milestone on the subject that Li et al. [25] found a positive correlation between the Systemic Lupus Erythematosus Disease Activity Index (SLEDAI) and the amount of *Streptococcus, Campylobacter* and *S. anginosus* in the fecal sample of SLE patients.

### 2.2. Dysbiosis in SLE Mice

Studying the microbiota in animal models, research has found some differences with humans and even between the different animal models (Table 2). Focusing on animal genetic models, changes have been described in the composition of gut microbiota during the course of the pathology; finding higher diversity in NZB/WF1, MRL/Mp-Faslpr (*lpr*) and SNF1 and toll-like receptor (TLR)-7.1 when the SLE had already set and even in the early stage of SLE [26,28]. Recently, no significant changes between female NZB/WF1 mice (33 weeks old) and age-matched control mice were observed regarding microbial richness, diversity, and evenness [29]. Regarding the F/B ratio, consensus has not been reached because some authors have found this parameter reduced [20] but others have not found any changes in NZB/WF1 or *lpr* mice [26,29].

Broadly, in the NZB/WF1, *lpr* and SNF1 and TLR-7.1 models, at the phylum level, there are changes present that are similar to those in human patients, namely elevated *Bacteroidetes* and reduced *Firmicutes.* Nevertheless, the main changes happen within the sublevel categories as family and genera. At the family level in the *lpr* mouse model, a decrease in *Lactobacillaceae* and a higher abundance of *Rikenellaceae*, *Desulfovibrionaceae*, *Ruminococcaceae*, *Lachnospiraceae*, or *Streptococcaceae* has been found [19,28]. These alterations are usually associated with changes in several genera such as *Tenericutes, Mollicutes, Butyrivibrio,* and *Roseburia*, which were enhanced in that model. Conversely, other genera were reduced, such as *Lactobacillus* or *Bifidobacterium* [28]. Furthermore, it has been found to modulate the genus *Anaerostipes*, which was negatively correlated with lupus activity [26]. *Anaerostipes* is able to maintain the gut health state due to the capacity to produce butyrate.

Furthermore, of these changes in the lupus-prone mouse model SNF1, authors have described some similar alterations described in other animal models such as the abundance of *Rikenellaceae* or *Lachnospiraceae* [1]. However, others have found differences in this model between pre-disease and disease stages, showing elevated *Lactobacillus*, which is usually decreased in other animal models and in humans. Other genera such as *Clostridium*, *Dehalobacterium*, *Oscillospira*, *Dorea* and *Bilophila* may also be elevated [26]. 

Another main animal model of SLE is NZB/WF1, in which the microbiota suffers alterations similar to *lpr* and humans at the phylum, family and genus levels. NZB/WF1 mice displayed a higher abundance of *Lactobacilli* in the gut microbiota, which may be associated with more severe clinical signs, especially the impairment of systemic autoimmunity and renal functions [26]. In *lpr* mice, some bacteria from the genus *Anerostipes* such as *Akkermansia muciniphila* have been significantly decreased from pre-disease to disease stage and also bacteria from the genus *Lactobacillus* or *Bifidobacterium* appear reduced [32]. Recently, in hypertensive female NZB/WF1, we found that the gut microbiota of these mice had a significantly higher abundance of *Pedobacter*, *Lactobacillus*, and *Prevotella* than the age-matched control group, without changes in other genera such as *Bifidobacterium* [29].

In recent years, a new model has been described, the TLR-7.1 model, and the microbiota has been analyzed by Zegarra-Ruiz et al. [30]. The alterations found are similar to those found in the other animal models. The microbiota is characterized by an increase in the families *Coriobacteriaceae* and *Rikenallecea*, and a reduction in *Clostridaceae*. At the genus level, the tise in *Prevotella* and *Desulfovibrio*, and the decrease in the abundance of *Turicibacter, Bifidobacterium*, *Coprobacillus and Anaerostipes* stand out. Finally, they found an increase in the species *Lactobacillus reuteri*, which was also found in mesenteric lymph nodes (MLNs), spleen and liver, linking its translocation to the evolution of SLE.

### 2.3. Gut Dysbiosis in Lupus Is Linked to Leaky Gut, Changes in Immune Cell Populations, and Cardiovascular Complications

Besides these changes, in gut microbiota from patients and animal models of SLE an alteration in the epithelium of the intestinal barrier characterized by an impairment in junction proteins like occludin, zonulin-1 and claudin and an increase in intestinal permeability, measured through fluorescein isothiocyanate (commonly referred to as FITC)-dextran, takes place [19]. Researchers are linking these alterations with the course of SLE because some bacteria and their structural components or products from bacterial metabolism might be able to cross the intestinal epithelium and reach blood and even several organs.

Recently, Manfredo-Vieira et al. [31] have shown how *Enterococcus gallinarum* is able to translocate from bowel to liver and activate the production of anti-dsDNA antibodies through TLR-7/8 activation in genetically predisposed hosts ((NZW × BXSB) F1 mice, and SLE patients). In fact, antibiotic treatment prevented mortality in this animal model, suppressed the growth of *E. gallinarum* in tissues, and eliminated pathogenic autoantibodies and T cells. In this fashion, Katz-Agranov et al. [32] found a negative correlation between gut levels of *Synergistetes* and plasma anti-dsDNA antibody titers and IL-6 levels. IL-6 can promote an increase in both the differentiation of T-helper (Th) 17 and in the production of IL-17a, which is an important driver of autoimmunity in SLE [33,34]. In relation to this, in in vitro studies, the fecal microbiota of SLE patients was a stronger inducer of Th17 [35].

Besides the modulation of anti-dsDNA antibody levels, bacteria could modulate the immune system in other ways, inducing beneficial or harmful effects in the course of SLE. Other possible mechanisms to modulate the immune system could be induced by the components of bacteria like lipopolysaccharide (LPS), which is derived from the wall of Gram-negative bacteria belonging to *Bacteroidetes* (this phylum is elevated in the gut microbiota of SLE mice), and thus, through TLR-4 activation, inducing the production of proinflammatory cytokines such as TNF-α, IL-6 and type I interferons (IFN-α, IFN-γ) that are elevated in SLE patients and also in animal models of SLE [36]. IFN-α could also be induced through TLR-7, a high-risk locus for excessive activation of RNA sensing, which is overexpressed in SLE [31]. This high level of IFN-α is able to induce endothelial dysfunction in SLE patients [37]. Inflammatory cytokines interact with important blood pressure regulatory systems, such as the renin–angiotensin system and the sympathetic nervous system [38]. The role of IL-6 has not specifically been examined in SLE hypertension. TNF-α has also been shown to be elevated in the serum of SLE patients and can correlate with disease activity [39]. Etanercept, a clinically available recombinant TNF-α receptor that reduces the biological activity of TNF-α, reduces mean arterial pressure in a female mouse model of SLE, suggesting that TNF-α mechanistically contributes to the development of hypertension [40].

TLR4 activation also contributes to increased blood pressure and low-grade vascular inflammation displayed by spontaneously hypertensive rats [41]. In fact, TLR4^−/−^ mice demonstrated full blood pressure protection against chronic endothelial nitric oxide synthase (eNOS) blockade-induced hypertension [42]. Bacterial LPS stimulates and increases the expression of TLR4 in the vasculature, which resulted in increased NADPH oxidase-dependent superoxide production, inflammation, and endothelial dysfunction [43,44]. Thus, enhanced TLR4 activation might be linked to the development and maintenance of hypertension in SLE. In fact, elevated plasma LPS levels have been previously described in both SLE patients [45,46,47] and hypertensive SLE mice [29].

In addition, some bacteria produce short-chain fatty acids (SCFAs), which are able to activate free fatty acid receptors with beneficial [48] or deleterious effects in the pathology. *Clostridium* and *Lachnospiraceae* are both producers of butyrate, which through the activation of G-protein-coupled receptor (GPR)-109a promotes differentiation of regulatory T cells in the colon, spleen, and lymph nodes and also IL-18, hence being capable of suppressing inflammation [49]. On the other hand, acetate and propionate seem elevated in stool samples of SLE patients, but Rodríguez-Carrio et al. [50] did not find differences in butyrate levels. These alterations in the production of bacterial by-products could be derived from changes in some metabolic pathways. The glycan degradation pathways are slightly overrepresented in the microbiota from SLE patients, likely due to the higher abundance of *Bacteroidetes* in these patients [51]. The treatment with vancomycin showed downregulated LPS biosynthesis [52].

Currently, researchers are seeking how to modulate the gut microbiota to improve this disease prognosis. According to this, certain bacteria could be correlated with the stages of remission in the course of the pathology or even with modifications carried out in some treatments. Katz-Agranov et al. [32] proved treatment with retinol decreased *Erysipelotrichaceae* and increased *Lachnospiraceae* and *Rikenellaceae*, restoring some changes observed in lupus. The treatment with dexamethasone or prednisone [53] (glucocorticoids widely used in patients with SLE) was able to increase the alpha diversity (Shannon index), which may lead to a more stable community [26]. The prednisone treatment was able to reduce the phyla *Proteobacteria* and *Deferribacteres.* The modulation of the gut microbiota induced several changes at the genus level, characterized by an increase in *Prevotella* and *Anaerostipes* and a decrease in *Rikenella*, *Mucispirillum*, *Oscillospira*, and *Bilophila* [53]. Antibiotic treatment with vancomycin was able to remove *Clostridiales* and *Bacteroidales*, while increasing *Lactobacilli* such as *L. rhamnosus* and *L. reuteri* [19].

**Figure 2:**
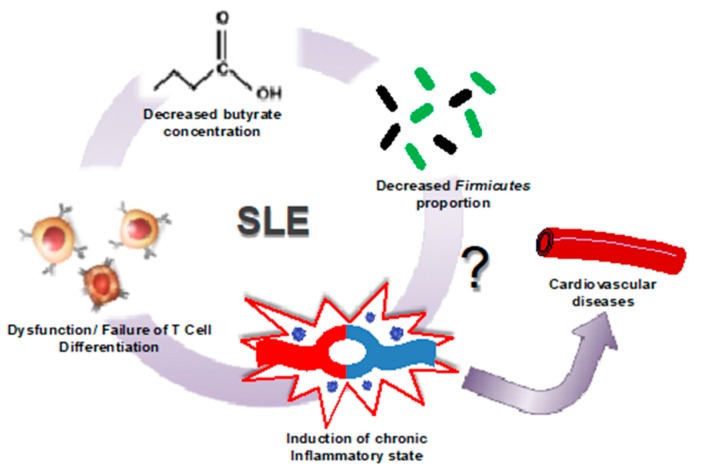
Microbiome shift in SLE induces cardiovascular risk. In patients with SLE, it has been observed that a decrease in the population of butyrate-producing *Firmicutes* leads to a decrease in butyrate production. This decreased production of butyrate leadsto the failure of T cells to properly differentiate in lupus patients, resulting in the induction of the chronic inflammatory state representative of this disease (modified from [44]).

In summary, the evidence is suggesting that the renal and cardiovascular complications associated with SLE are a consequence of an immune response aggravated by a high multitude of antigen-presenting bacteria such as *Lachnospiraceae*, *or Bacteroidetes,* and a decrease in *Firmicutes* abundance within the gut microbiome, resulting in lower butyrate levels, T cell dysfunction, and the onset of chronic inflammation and cardiovascular risk (Figure 2) [54]. However, it is not clear whether intestinal dysbiosis in SLE is cause or consequence [35]. Current evidence suggests that intestinal microbes could be involved in the initiation and amplification of autoimmune diseases, such as SLE. Despite recent progress in understanding how these microbes influence the pathophysiology of lupus, studies are still limited. Recently, an association between gut microbiota, free fatty acid serum pool, and biomarkers of endothelial activation in lupus patients has been stablished, thus emphasizing the systemic effect of the gut microbiota in this condition [50]. However, there is no direct link between gut dysbiosis and endothelial dysfunction and hypertension in SLE. It is possible that reducing Th17 polarization by increasing butyrate-producing bacteria, or decreasing endotoxaemia by improving gut integrity, or reducing autoantibodies production by both reducing commensal bacteria containing an RNA-binding autoantigen, Ro60 structural homologs [55], or by preventing translocation of specific bacteria, such as *Enterococcus gallinarum* [31], or *Lactobacillus reuteri* [30] to secondary lymph organs and liver, could prevent cardiovascular complications in SLE.

## 3. SLE and Probiotics

Due to the aforementioned discoveries, and especially because of the depletion of *Lactobacillus* and *Bifidobacterium* in the gut microbiota present in the lpr SLE models, researchers thought that treating lupus patients with bacterial supplementation (Table 3), such as different strains of *Lactobacillus* that had already shown some effects in other autoimmune diseases [56,57], could help ameliorate the disease symptomatology. In fact, Mu et al. [19] showed that supplementation with *Lactobacillus* spp. in *lpr* mice displayed a striking effect mitigating lupus nephritis and prolonging survival. These effects are linked to a reduced plasma anti-dsDNA levels induced by probiotic consumption.

These microorganisms that in adequate amounts can provide a health benefit to the treated host receive the name of probiotics. Although certain limitations must be applied to this term according to the last panel of the International Scientific Association for Probiotics and Prebiotics (ISAPP), gathering only products that deliver live microorganisms with a suitable viable count of well-defined strains with a reasonable expectation of delivering benefits for the wellbeing of the host under this name [58]. Previous evidence showed that probiotic supplementation could be effective in the prevention and treatment of cardiovascular disease in obese postmenopausal women. In fact, supplementation with multispecies probiotic Ecologic^®^ Barrier favorably modified both functional and biochemical markers of vascular dysfunction, reduced systolic blood pressure [59], and improved cardiometabolic parameters [60]. These protective effects are related to lower plasma LPS levels found in women with probiotic supplementation. However, whether probiotic consumption improves endothelial dysfunction in SLE patients is still unknown.

The physiological responses to different probiotics have proven to be consistently diverse. Typically, these microorganisms trigger immune shifts that effectively decrease the inflammatory response, thus, ameliorating the symptomatology of autoimmune diseases. Some of the most commonly observed mechanisms in the treatment of SLE are related to the modulation of Th17 and T regulatory (Treg) lymphocyte populations [61]. Nonetheless, other less studied mechanisms are also involved.

According to Mardani et al. [61], the administration of the probiotic *Lactobacillus delbrueckii* subsp. *Lactis* PTCC 1743 to a pristane-induced SLE mice model was able to improve the disease symptoms, decreasing Th17 populations and the expression of one of its main cytokines, IL-17a, well known as crucial elements in the development and maintenance of inflammation. In addition to this, another probiotic microorganism, *Lactobacillus rhamnosus* ATCC 9595, showed the capacity to modulate retinoic acid receptor-related orphan receptor gamma (RORγ), a transcription factor involved in the maturation of Th17 lymphocytes, which would explain the ability to decrease this lymphocytic population. Both probiotics were also able to reduce Th1 populations and its cytokine IFN-γ, which is regarded as one of the main mechanisms involved in the generation of the inflammatory response.

On the other hand, *Lactobacillus reuteri* GMNL 263 displayed a different pathway in the genetic murine model of SLE NZB/W F1 [62]. This microorganism can increase Treg lymphocyte expression and its transcription factor forkhead box P3 (FoxP3) levels. These cells are responsible for the regulation of the pro-inflammatory lymphocytes cited above, and with a marked anti-inflammatory character. Aside from this, levels in common pathogen-associated molecular pattern (or PAMPs) receptors such as TLR-4, TLR-5, TLR-7 and TLR-9 that mediate in the development of inflammation were also reduced in the liver with the probiotic treatment, as well as an increase in antioxidant activity. The aforementioned changes in TLRs and oxidative stress were also detected in similar experiments with probiotics *Lactobacillus paracasei* GMNL 32 (GMNL-32) and *Lactobacillus reuteri* GMNL 89, although the effects of *Lactobacillus reuteri* GMNL 263 on Treg expression were not present in those cases. Furthermore, in these three treatments, through the suppression of nuclear factor kB (NF-κB) and the mitogen-activated protein kinase signaling pathways, there was a reduction in hepatic pro-inflammatory cytokines IL-1β, TNF-α and IL-6 [63,64]. Interestingly, GMNL-32 treatment reduced left ventricular hypertrophy in this genetic model of lupus.

Furthermore, dendritic cells exposed to SLE microbiota enriched with *Bifidobacterium bifidum* LMG13195 tended to, when co-cultured with naïve T cells, lessen the activation of these lymphocytes when compared to normal SLE microbiota in vitro. In the same conditions but using *Ruminococcus obeum* DSM25238 and *Blautia coccoides* DSM935 instead of the *Bifidobacterium* strain, after recovering the T cells resulting from the co-incubation, decreased Th17/Th1 ratios were observed, changing the inflammation pattern when compared to normal SLE microbiota. However, when studying IFN-γ and Il-17a expression levels, no significant differences were observed [35].

**Figure 3:**
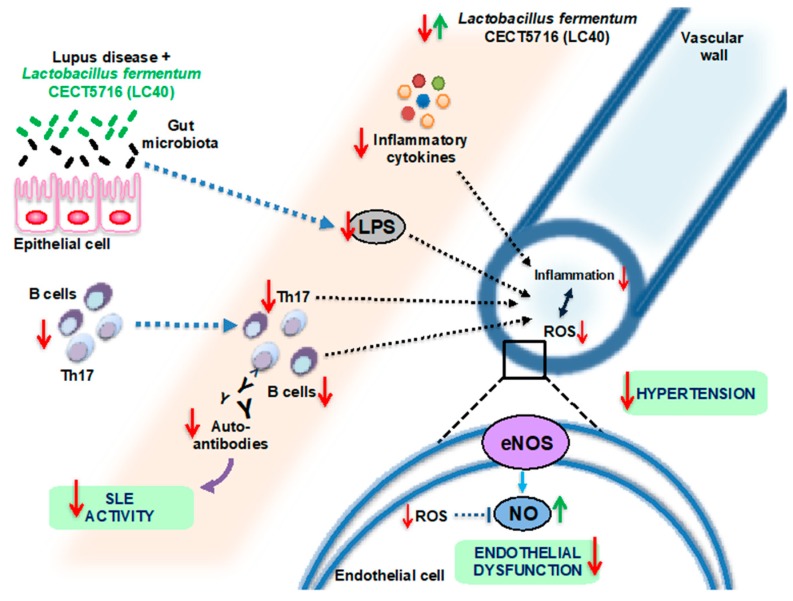
Proposed mechanism by which *Lactobacillus fermentum* CECT5716 (LC40) administration could prevent SLE activity, endothelial dysfunction and hypertension in the NZB/WF1 model (based on data from publication no. [29]).

Female NZB/WF1 mice have several characteristics consistent with human SLE, including immune complex deposition in the glomerulus, dsDNA autoantibodies, albuminuria and, importantly, endothelial dysfunction and hypertension [65]. Chronic *Lactobacillus fermentum CECT5716* (LC40) consumption in female NZB/WF1 mice was able to increase the *Bifidobacterium* count in the gut. LC40 reduced lupus disease activity and splenomegaly in SLE mice. It also improved gut barrier integrity, reducing LPS plasma levels, which in turn reduced immune activation, shown by a decrease in T and B cells in MLN and pro-inflammatory cytokines such as IL-17a, IFN-γ, TNF-α and IL-21 in plasma. As the probiotic prevented the development of the pro-inflammatory response, complications associated with SLE such as cardiac and renal hypertrophy were averted. Moreover, high blood pressure and impaired vascular endothelium-dependent vasodilation were prevented by *L. fermentum* as a result of reduced eNOS phosphorylation at the inhibitory site Thr^495^, and decreased NADPH oxidase activity, commonly considered as the main source of reactive oxygen species in the vascular system, which improved the bioavailability of nitric oxide [29] (Figure 3).

Therefore, adjustment of the SLE microbiome via dietary intervention has shown attenuation of SLE symptoms and, in one case, of its cardiovascular complications, marking this strategy as a possible novel therapeutic approach to this disease.

As seen with the examples above, despite having a clearer picture of the elements involved in the effect of probiotics in SLE, especially the role of Treg and Th17 modulation, a better understanding of the underlying mechanisms of probiotics is needed, which would not only allow for better targeting and selection of microorganisms but could also cast some light on the development and causes of SLE and its renal and cardiovascular complications.

## 4. Conclusions

This review opens new possibilities to the prevention SLE and its renal and cardiovascular disorders and the modulation of the gut microbiota through the administration of some probiotic strains. However, caution is advised when extrapolating these findings to humans because of the possible differences in the behavior of the animal and human gut microbiota. In fact, the relative abundance of *Lactobacillales* appears to be normal in SLE patients in remission [18], which might alter the possible applicability of the *Lactobacillus* treatment to clinical practice in humans. Although not a diet in itself, there is a common belief that probiotic-containing fermented foods modify the gut microbiota and confer host health benefits. However, fermented foods rarely contain adequate amounts of specific probiotic organisms. Moreover, there is limited evidence for the role of probiotics as modulators of the human gut microbiota, and recent data suggest that even supplemental quantities of probiotics exert limited effects on human gut ecology [66]. On the other hand, the composition of gut microbiota in a large population of hypertensive SLE patients is unknown.

The answer to these questions could clarify not only the mechanisms involved in the protective effects of specific probiotic bacteria but also their potential in human SLE treatment. Taking into account that the pathophysiological mechanism involved in disorder development in these patients may vary and that the mechanisms of improvement in SLE disease by specific probiotics are unknown, it is worth investigating whether we can choose a specific probiotic strain to gain benefits in a particular SLE patient. Taken together, preclinical and clinical evidence indicates that further research is needed to evaluate the safety profile before any of these probiotics can be marketed for the clinical treatment of hypertension, metabolic, and vascular complications in SLE. However, we suggest that beneficial bacteria, such as *L. fermentum*, capable of reducing dysbiosis, improving gut barrier function and reducing endotoxemia might prevent vascular complications in SLE patients. Unfortunately, when consulted for this review, clinicaltrials.gov does not yet include any trial on SLE patients with probiotics.

## Figures and Tables

**Table 1:** Gut microbiota shifts in systemic lupus erythematosus (SLE) patients.

Patients	Ratio F/B	α-Diversity	Phylum	Family	Genus	Species	Reference
Women C 49.2 ± 10.7 years 20 patients	↓F/B	No change	↓*Firmicutes* ↓Tenericutes ↑*Bacteroidetes*	↓*Lachnospiraceae* ↓*Ruminococcaceae*			[18]
Women A 46.0 ± 1.8 years 35 patients	↓F/B	↓PD_whole_ tree ↓Observed species	↓*Firmicutes* ↑*Bacteroidetes* ↑*Actinobacteria* ↑*Proteobacteria* ↑Fusobacteria	↑*Bacteroidaceae* ↑*Prevotellaceae* ↑*Rikenellaceae*	↓*Pseudobutyvibrio* ↓*Dialister* ↓*Bifidobacterium* ↑*Rhodococcus* ↑*Eggerthella* ↑*Klebsiella* ↑*Prevotella* ↑*Flavonifractor* ↑*Eubacterium*		[24]
Women A 37.46 ± 14.17 years 40 patients	↓F/B	↓Chao Richness ↓PD_whole_ tree ↓Observed species	↓Tenericutes	↑*Streptococcaceae* ↑*Lactobacillaceae* ↑*Megasphaera*	↓*Mollicutes* ↓*RF39* ↓*Faecalobacteriu*, ↓*Cryptophyta* ↓*Roseburia*	↑*Streptococcus anginosus* ↑*Lactobacillus mucosae* ↑*Veinella dispar*	[25]
Women 3 AA (42.33 ± 13.39 years), 7 C (49.42 ± 8.51 years) Men 3 C (33 ± 6.57 years) 1 AA (29) 14 patients	Not change		↑*Proteobacteria*		↑*Blautia*		[26]
Women 10 C (38.3 ± 4.32 years) 13A (38.3 ± 4.32 years) 16 AA (46.69 ± 4.33 years) 19 WH (44.84 ± 3.5 years) 3 BH (43 ± 9.57 years) 61 patients		↓Chao Richness		↓*Ruminococcaceae*	↑*Blautia*	↓*Ruminococcus gnavus* ↓*Bacteroides uniformis*	[27]

C, Caucasian; AA, Afro-American; A, Asian; WH, White Hispanic; BH, Black Hispanic; Age (means ± SD); F/B, Firmicutes/Bacteroidetes. ↓Reduction, ↑Increase.

**Table 2:** Gut microbiota shifts in different lupus animal models.

	Ratio F/B	α-Diversity	Phylum	Family	Genus	Species	Reference
NZB/WF1	↓F/Bor no change	↑α-diversity or no change	↓*Firmicutes* ↑*Bacteroidetes*		Pre-SLE ↓*Bifidobacterium* ↑*Lactobacillus* ↓*Lactobacillus* High severity ↓*Anerostipes*		[26] [28] [29] [30]
MRL/lpr	↓F/Bor no change	↑α-diversityor no change	↓*Firmicutes* ↑*Bacteroidetes*	↓*Lactobacillaceae* ↑*Rikenellaceae* ↑*Desulfovibrionacea* ↑*Ruminococcaceae* ↑*Lachnospiraceae* ↑*Streptococcaceae*	↓*Lactobacillus* ↓*Bifidobacterium* ↑*Tenericutes* ↑*Mollicutes* ↑*Butyrivibrio* ↑*Roseburia*		[19] [26] [28] [30]
SNF1	↓F/Bor no change	↑α-diversity	↓*Firmicutes* ↑*Bacteroidetes*	↑*Rikenellaceae* ↑*Lachnospiraceae*	Pre-SLE ↓*Lactobacillus* SLE ↑*Lactobacillus* ↑*Clostridium* ↑*Dehalobacterium* ↑*Oscillospira* ↑*Dorea* ↑*Bilophila*		[1] [26]
TLR-7.1	↓F/B	↑α-diversity	↓*Firmicutes* ↑*Bacteroidetes*	↓*Clostridaceae* ↑*Coriobacteriaceae* ↑*Rikenallecea*	↓*Turicibacter* ↓*Bifidobacterium* ↓*Coprobacillus* ↓*Anaerostipes* ↑*Prevotella* ↑*Desulfovibrio*	↑*Lactobacillus reuteri*	[31]

F/B, Firmicutes/Bacteroidetes; SLE, systemic lupus erythematosus. ↓Reduction, ↑Increase.

**Table 3:** Probiotic effects in different lupus animal models.

Probiotic	Model	Observed Effects	Reference
*Lactobacillus delbrueckii* subsp. lactis PTCC 1743	Pristane-induced murine model	↓Th17 ↓IL-17a ↓Th1 ↓IFN-γ	[61]
*Lactobacillus rhamnosus* ATCC 9595	Pristane-induced murine model	↓RORγ ↓Th17 ↓Th1 ↓IFN-γ	[61]
*Ruminococcus obeum* DSM25238	In vitro	↓Th17/Th1 ratio	[35]
*Blautia coccoides* DSM935	In vitro	↓Th17/Th1 ratio	[35]
*Lactobacillus reuteri* GMNL 263	NZB/W F1	↑FoxP3 ↑Treg ↓TLR-4 ↓TLR-5 ↓TLR-7 ↓TLR-9	[62]
		↓IL-1β ↓TNF-α ↓IL-6	[63]
*Bifidobacterium bifidum* LMG13195	In vitro	↓T lymphocytes activation	[35]
*Lactobacillus fermentum* CECT5716	NZB/W F1	↓B and T lymphocytes↓IL-17a ↓IFN-γ ↓TNF-α ↓IL-21	[29]
*Lactobacillus reuteri* GMNL 89	NZB/W F1	↓TLR-4 ↓TLR-5 ↓TLR-7 ↓TLR-9	[62]
		↓IL-1β ↓TNF-α ↓IL-6	[48]
*Lactobacillus paracasei* GMNL 32	NZB/W F1	↓TLR-4 ↓TLR-5 ↓TLR-7 ↓TLR-9	[62]
		↓IL-1β ↓TNF-α ↓IL-6	[63]

FoxP3, forkhead box P3; IFN, interferon; IL, interleukin; RORγ, RAR-related orphan receptor gamma; Th, T-helper; TLR, toll-like receptor; TNF-α, tumor necrosis factor alpha; Treg, T regulatory. ↓Reduction, ↑Increase.
